# Test selection for antibody detection according to the seroprevalence level of Schmallenberg virus in sheep

**DOI:** 10.1371/journal.pone.0196532

**Published:** 2018-04-27

**Authors:** Srđan Pejaković, Laëtitia Wiggers, Damien Coupeau, Nathalie Kirschvink, James Mason, Benoît Muylkens

**Affiliations:** 1 NAmur Research Institute for LIfe Sciences (NARILIS), University of Namur, Namur, Belgium; 2 Integrated Veterinary Research Unit, Department of Veterinary Medicine, Faculty of Sciences, University of Namur, Namur, Belgium; 3 School of Biochemistry & Immunology, Trinity College Dublin, the University of Dublin, Dublin, Ireland; Sciensano, BELGIUM

## Abstract

Schmallenberg virus (SBV), initially identified in Germany in 2011, spread rapidly throughout Europe causing significant economic losses in ruminant livestock. The ability to correctly detect emerging and re-emerging diseases such as SBV with reliable tests is of high importance. Firstly, we tested diagnostic performance, specificity, and sensitivity of three different assays used in SBV antibody detection using control sheep samples of determined status. After obtaining the results from the control samples, we assessed the potential of the assays to detect previously infected animals in field situations. The samples were investigated using IDEXX Schmallenberg virus Antibody Test Kit, ID Screen Schmallenberg virus Competition Multi-species ELISA and Serum Neutralisation Test (SNT). Analysis of control samples revealed that SNT was the most suitable test, which was therefore used to calculate concordance and test performance for the two other ELISA tests. To evaluate whether different assay performances had an impact under field conditions, sheep samples from two different contexts were tested: the emergence of SBV in Ireland and the re-emergence of SBV in Belgium. Comparing the results obtained from different assays to the non-reference standard assay SNT, we showed considerable differences in estimates of their sensitivity to detect SBV antibodies and to measure seroprevalence of the sheep flocks. Finally, a calculation of the number of randomly selected animals that needs to be screened from a finite flock, showed that SNT and ID.Vet are the most suitable to detect an introduction of the disease in low seroprevalence situations. The IDEXX ELISA test was only able to detect SBV antibodies in a higher seroprevalence context, which is not optimal for monitoring freedom of disease and surveillance studies.

## Introduction

Schmallenberg virus (SBV) was first identified in Germany in November 2011 [1]. It affects domestic and wild ruminants, in which it can cause abortions, stillbirths, or severe congenital malformations depending on the stage of embryological development of the offspring [2]. The ability of the virus to cross the placental barrier, and the effect on the adult animals and export restrictions, led to significant economic losses in European ruminant livestock from November 2011 to spring 2013 [3, 4, 5]. Flying insects of the *Culicoides* spp. have been identified as vectors that play a key role in the spread of SBV [6, 7]. Since its discovery, evidence of SBV infection in livestock has been found throughout Europe. A number of studies in 2016 showed a reoccurrence of SBV infections on the continent, leading to the conclusion that the virus became endemic. The virus arrived in Ireland in 2012, where the first case of SBV was diagnosed in October 2012 [8] and its effects were very evident in the southeast of Ireland [9]. In the 2013 lambing season in the East Wicklow area, reported losses were significant (up to 10% of affected lambs) and the typical deformed foetuses were observed, as well as some abortions. SBV spread rapidly across Europe, and Belgium has been identified as one of the first and most affected countries [10]. After initial SBV emergence in April 2011, massive spreading of viral infection occurred. The last case of SBV infection in Belgium was confirmed by PCR on an aborted foetus in March 2013 [11, 12]. Three years after the last reported outbreak of SBV, in April 2016, an abortion occurred in a two-year-old cow that previously had not been vaccinated, showing a new re-emergence of the virus in Belgium [13].

Correctly detecting emerging and re-emerging diseases such as SBV requires a reliable test that produces stable and consistent results. Therefore, the objectives of this study were to measure and compare the diagnostic performance including specificity and sensitivity of three tests used in SBV antibody detection. In order to do so, sheep reference samples of known status were submitted to three assays: IDEXX Schmallenberg virus Antibody Test Kit (IDEXX), ID Screen Schmallenberg virus Competition Multi-species ELISA (ID.Vet) and Serum Neutralisation Test (SNT). In addition, diagnostic performance measures, including Youden’s index (J) and Cohen’s kappa (κ) were calculated based on reference samples.

After obtaining results from the control sheep samples two questions were raised. How would these assays perform in the field conditions? Moreover, are there noticeable differences in their ability to detect previously SBV infected animals from the field samples? To address these questions, field sheep samples obtained from two independent situations were used to evaluate agreement between IDEXX test, ID.Vet test and SNT. Sheep serum samples obtained during the 2013 emergence of SBV in Ireland represented the first field situation, while the second was represented by serum samples from ewes and their lambs obtained during the 2016 re-emergence in Belgium. Positive and negative percentage of agreement, and Cohen’s kappa (κ) were calculated in order to evaluate overall discriminative abilities of diagnostic assays. It was done by comparing two screening ELISA tests in their performance and by measuring the degree of concordance with the SNT as the non-reference standard.

Finally, a hypothetical sample size calculation was used to estimate the random number of animals that should be tested using three assays in order to assess if the population is SBV infected in different theoretical seroprevalence situations.

## Materials and methods

### Samples and study design

#### Reference samples of known status

To correctly assess diagnostic performance, i.e., sensitivity and specificity of the three assays used in this study, control samples of known status were tested. Serum samples from ewes, collected from University of Namur’s sheep flock during the 2012 SBV re-emergence, with a clinical presentation of SBV infection (n = 45), and confirmed by PCR analysis, were used as true positives. 100% of animals that tested positive in PCR seroconverted in experimental conditions [14]. Serum samples collected from sheep in 2008 (n = 45), before the first SBV emergence, represented the true negatives. The information and results from assays performance obtained from the control samples were later used for precise determination of infection status of samples from the field situations.

#### Field samples obtained from two different situations

To assess the ability of the tests to detect previously SBV infected animals from field samples, sera were obtained from two different contexts. First, from Ireland, blood samples tested were taken in April 2013 from two areas of East Wicklow, Ashford and Rathdrum. Sixty-nine ovine blood samples in total were collected, 38 from Ashford and 31 from the Rathdrum area. Samples were collected from sheep flocks where congenital deformations were detected in January and February 2013. Thus, infection in ewes likely happened in autumn 2012, although the true infection status of the samples was not known. Second, from Belgium, sera from 22 ewes and 36 of their lambs were selected and sampled from the sentinel sheep flock (n = 420) from the University of Namur. For lambs, sera were sampled at 0 and 48 hours post-lambing (hpl), giving 72 serum samples in total. Samples obtained from ewes and from lambs 48 hpl represented positive samples, while samples from lambs at 0 hpl represented negatives. Samples were collected in September 2016.

#### Ethics statement

This study was conducted in accordance with Belgian and Irish law for animal protection and with the European Directive, 2010/63/EU. In Ireland, the blood samples were collected by the residence veterinary service according to the Irish and EU legislations. The Ethics committee of the University of Namur, (CEEXPANI, project n° 12/185) specifically approved this study.

### Serological analysis

#### IDEXX Schmallenberg virus Antibody Test Kit

Control and serum samples were analysed following the manufacturer’s recommendations. The optical density of the samples was analyzed in relation to the negative and the positive controls provided by the manufacturer (S/P %). Samples presenting an S/P percentage < 30% were considered negative, 30% ≤ S/P % < 40% were considered suspect, and greater than or equal to 40% were considered positive. The specificity and sensitivity of the IDEXX Schmallenberg virus Antibody Test Kit were reported to be 99.5% and 98.1%, respectively [15].

#### ID Screen Schmallenberg virus Competition Multi-species Test

Control samples and serum samples were analyzed following the manufacturer’s recommendations by using positive and negative controls provided by the manufacturer. For each sample, the relation to the negative control (S/N %) was calculated and samples presenting an S/N percentage less than or equal to 40% were considered positive, 40% < S/N % ≤ 50% as doubtful, and greater than 50% were considered negative. A specificity of 100% and a sensitivity of 97.6% were reported for the ID.Vet ID Screen Schmallenberg virus Multi-species Test [16].

#### Serum Neutralisation Test

The Serum Neutralisation Test (SNT) is a serological test used to detect the presence of functional antibodies that prevent infectivity of a virus. In-house SNT was performed as follows [14]. Baby hamster kidney-21 cells (BHK-21) were grown in Glasgow’s MEM medium (GMEM, Life Technologies), supplemented with 10% of foetal bovine serum (FBS, decomplemented for 30 minutes at 56°C), penicillin-streptomycin (PS, 10000 U/ml) and tryptose-phosphate broth (Sigma). 96-well plate was seeded with BHK-21 (4 × 10^4^ cells in 150 μl /well in total) in duplicates. A dilution plate was prepared by adding 120 μl of serum in first wells, from where eight 2-fold dilutions were made in 60 μl of MEM medium (Lonza). From the last wells, 60 μl of dilution was discarded leaving 60 μl of final volume. A virus isolate SBV-BH80/11–4 (Friedrich-Loeffler-Institute, Germany) was prepared to work concentration using 1% of decomplemented FBS, 2% PS and added to the wells of the 96-well plate. 60 μl of the prepared virus was added to each well of the dilution plate containing diluted serum samples to obtain a tissue culture infectious dose 50 (TCID50) between 100–200 and incubated overnight (O/N) at 37 °C with 5% CO2. After O/N incubation, 50 μl of virus-serum mixture was added into appropriately labelled wells of 96-well plate containing BHK-21 cells and incubated for two hours at 37 °C. After two hours, 100 μl of GMEM medium was added into the wells. Cells were then incubated for four days at 37 °C with 5% CO2. Afterwards, the medium from the wells was discarded and 100 μl of crystal violet solution (Merck) was added to each well. The plates were incubated at room temperature for 10 minutes. Next, the wells were rinsed under running cold water until excess crystal violet was removed. The plates were dried on tissue paper and the presence or absence of cytopathic effect in each well was determined. The data were expressed as log_2_ of the dilution of serum that neutralised 50% of cell lysis in the inoculated wells (ED50). Results were considered positive if log_2_ ED50 was higher than two. For validation of SNT virus back titration was performed, where TCID50 of 100–200 per 120 μl represented validated test.

### Data analysis and test evaluation

#### Seroprevalence, specificity, and sensitivity

Seroprevalence was defined as the number of animals in a population that tested positive for SBV antibodies based on serum samples. It was calculated as a percentage of the total tested samples. Specificity and sensitivity were calculated to evaluate the three diagnostic assays used in this study. For each analysis, 95% confidence intervals (CI) were calculated using the Clopper-Pearson exact method.

#### Youden’s index and Cohen’s Kappa

Youden’s index (J) was used to evaluate the overall discriminative abilities and diagnostic performances of the three diagnostic assays in question on the control samples. The possible range of values is between -1 and 1. A zero value indicates a useless assay, and a value of one indicates a perfect diagnostic assay. The test performance is evaluated by considering both sensitivity and specificity and was calculated as follows:

J=Sensitivity+Specificity−1

Cohen’s kappa (κ) was used to measure the degree of concordance among ELISA tests and SNT used in the study resulting in a score of homogeneity in the ratings given by different assays. κ values can range from −1 to 1, where zero represents no agreement. First, the proportion of units with agreement (p) was calculated:

$$p=\frac{n{}^{\circ}\;of\;samples\;in\;agreement}{total\;n{}^{\circ}\;samples}$$

Then the proportion of units that would be expected to agree by chance (p_e_):

$$p_{e}=the\;probability\;that\;the\;tests\;randomly\;are\;positive\;+\;the\;probability\;that\;the\;tests\;randomly\;are\;negative$$

Then, Cohen’s kappa was calculated as follows:

$$\kappa =\frac{\;(p-p_{e})\;}{\left(1-p_{e}\right)}$$

The standard error of kappa (SE) was calculated by *SE* = $\sqrt{\frac{p\;(1-p)}{n\;{\left(1-p_{e}\right)}^{2}}}$

If the value of κ is between 0–0.20, the level of agreement of the two tests is none and the percentage of reliable data is 0–4%. A κ value from 0.21–0.39 shows a minimal level of agreement (with 4–15% of reliable data) and from 0.40–0.59, the level of agreement is weak (15–35%). If the values of κ are from 0.60–0.79, the level of agreement is moderate (35–63%) and from 0.80–0.90, the agreement is strong (64–81%). Finally, the level of agreement for a κ value ranging from 0.91–1 is almost perfect or perfect (82–100%).

#### Positive and negative percentage of agreement

Working with a non-reference standard, such as SNT, is not appropriate for calculating relative sensitivity and specificity of the two ELISA tests used to detect SBV antibodies in the field samples. Because of that, the positive percentage of agreement (PPA) and negative percentage of agreement (NPA) were used to describe the level of concordance between the SNT and the two ELISA tests. The PPA and NPA are based on the same calculations as sensitivity and specificity, but they reflect the non-reference standard.

#### Sample size calculation for SBV antibodies detection in a finite sheep population using different theoretical seroprevalence situations

Hypothetical sample size calculation was used to estimate the random number of animals that should be tested using three assays in order to assess if the population is SBV infected. To do so, different theoretical seroprevalence situations (1–99%) were examined. A modified hypergeometric distribution was used as described before [17], which calculated the exact probability of detecting diseased animals considering both imperfect tests (different assay’s sensitivities and specificities obtained from the control testing) and finite sheep population (n = 200). According to Eurostat information for 2013, the average number of sheep in European Union holdings was 115, thus it was decided that a finite sheep flock of 200 should represent realistic population size.

## Results

### SNT as a reference test to identify SBV infected sheep

**Fig 1:**
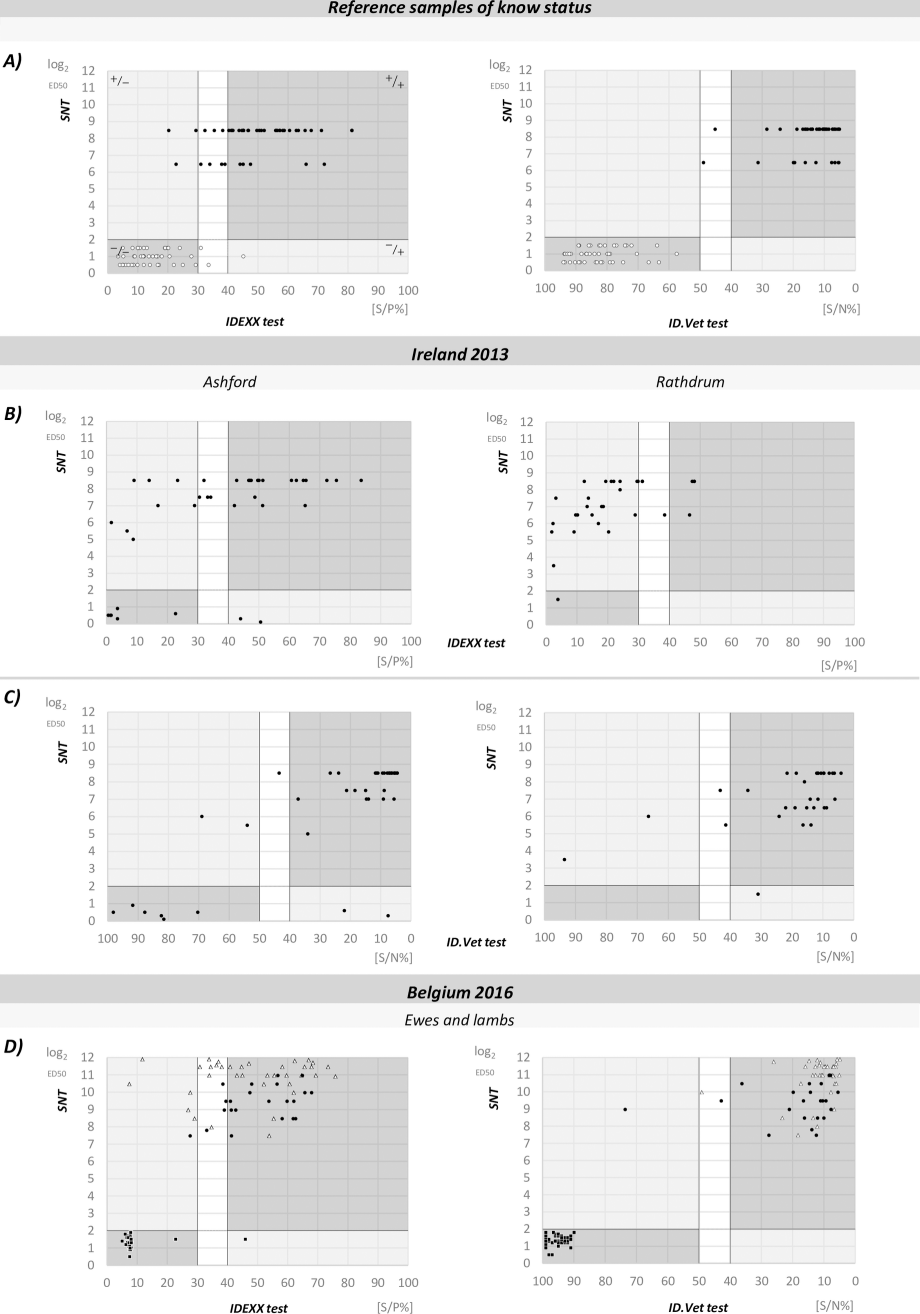
Different sensitivities and agreements between Serum Neutralisation Test (SNT), IDEXX Schmallenberg virus Antibody Test Kit (IDEXX test) and ID Screen Schmallenberg virus Competition Multi-species ELISA (ID.Vet test). **(A)** Results obtained from reference samples of known status. Black dots represent positive and white dots negative controls. **(B)** Different sheep populations (black dots) from Ireland (Ashford and Rathdrum area) tested with IDEXX test and their diagnostic performance compared to SNT. **(C)** Same sheep populations (black dots) from Ireland (Ashford and Rathdrum area) tested with ID.Vet test and their diagnostic performance compared to SNT. **(D)** Sheep samples collected after re-emergence in Belgium (ewes and lambs) are shown. Circles—representing ewes, squares—lamb sera collected at 0 hours post-lambing (hpl) and triangles—lamb sera collected at 48 hpl. Dark lines represent positive thresholds. Quadrants of the first graph are marked with + or–symbols representing SNT tested value/ELISA tested value and refer to all of the graphs. The unshaded area represents the suspect/doubtful range for ELISA tests according to the manufacturers.

To assess sensitivity and specificity of the three assays used to detect SBV antibodies, positive and negative controls of known status were tested. From 90 samples in total, 45 serum samples collected from ewes with a clinical presentation of SBV (malformed offspring) were used as the true positives (confirmed by PCR analysis), while 45 serum samples collected from ewes before the first SBV emergence in Europe represented the true negatives (Table 1). From all three assays tested, only SNT read samples with sensitivity and specificity of 100% (CI = 92%-100%) with diagnostic performance of 1 and perfect agreement (Fig 1A).

**Table 1:** Diagnostic performances of three serological tests used to detect Schmallenberg virus antibodies from samples of known status with inclusion of suspect/doubtful in the negative results (a) and in the positive results (b).

**a) Suspect/doubtful included in the negative results**					
	**SNT**		**IDEXX**		**ID.Vet**
**Infected (n = 45)**					
*True positive	45		35		43
False negative	0		10		2
**Uninfected (n = 45)**					
**True negative	45		44		45
False positive	0		1		0
**Seroprevalence (%)**	50 (39–61)		40 (30–51)		48 (37–59)
**Sensitivity (%)**	100 (92–100)		78 (63–89)		96 (85–99)
**Specificity (%)**	100 (92–100)		98 (88–100)		100 (92–100)
**J**	1		0.75		0.96
**κ**^†^	1 (0.105)		0.75 (0.103)		0.96 (0.105)
**b) Suspect/doubtful included in the positive results**					
	**SNT**		**IDEXX**		**ID.Vet**
**Infected (n = 45)**					
*True positive	45		42		45
False negative	0		3		0
**Uninfected (n = 45)**					
**True negative	45		42		45
False positive	0		3		0
**Seroprevalence (%)**	50 (39–61)		58 (47–68)		50 (39–61)
**Sensitivity (%)**	100 (92–100)		93 (82–99)		100 (92–100)
**Specificity (%)**	100 (92–100)		93 (82–99)		100 (92–100)
**J**	1		0.86		1
**κ**^†^	1 (0.105)		0.86 (0.105)		1 (0.105)

Abbreviations: SBV—Schmallenberg virus, J—Youden’s index, κ - Cohen’s Kappa

*Serum samples (n = 45) collected during 2012 re-emergence from sheep with a clinical presentation of Schmallenberg virus (SBV) infection (malformed offspring) and confirmed by PCR analysis in the sampled blood represented true positives.

**Serum samples (n = 45) collected from sheep in 2008 before first SBV emergence in Europe represented true negatives. Percentage range of exact 95% confidence intervals (CI) is shown between the brackets.

^†^ For each κ value, the standard error is indicated between the brackets.

However, the IDEXX test read 16% and 4% of positive and negative reference samples as a suspect, respectively, and the ID.Vet test considered two (4%) reference samples as doubtful (S1 Table). In order to avoid biased estimation of the sensitivity and specificity, the results for the ELISA tests were reported under two scenarios.

The first scenario, where all suspect/doubtful reads were included in negative results, showed reduced sensitivities for IDEXX and ID.Vet tests of 78% (CI = 63%-89%) and 96% (CI = 85%-99%) respectively. This was reflected in reduced performance of 0.75 and moderate agreement for IDEXX test. For ID.Vet test performance was 0.96 with almost perfect agreement. However, specificities for IDEXX and ID.Vet tests were 98% (CI = 88–100) and 100% (CI = 92%-100%), respectively (Table 1A).

In the second scenario, where all suspect/doubtful reads were included in positive results, the IDEXX test correctly diagnosed 93% (CI = 82%-99%) of positive reference animals and yielded three false positives among the negative controls, with sensitivity and specificity of 93% (CI = 82%-99%). In addition, a diagnostic performance and strong agreement of 0.86 were obtained. The ID.Vet test recognized true positive and true negative samples with specificity and sensitivity of 100%, and a perfect diagnostic performance and perfect agreement (CI = 92%-100%) (Table 1B).

These results obtained from the reference material revealed that SNT had the best diagnostic performance among three assays, and it could be used as the non-standard reference test to define the true infection status of sheep samples (Fig 1A). Furthermore, the lack of sensitivity observed in reference material contrasted with the values reported by manufacturers, especially for the IDEXX test. The ID.Vet test scored better with results closer to reported values, and diagnostic performance and agreement closer to that of the SNT.

### Performance of three assays tested in the field contexts

The first field context consisted of sheep samples collected during 2013 SBV emergence in Ireland. Samples from two areas of East Wicklow (Ashford and Rathdrum) were tested. The SNT yielded 60/69 positive, with seroprevalence of 79% (CI = 63%-90%) in the Ashford area and 97% (CI = 83%-100%) in the Rathdrum area (Table 2, part I). However, in these two areas, the IDEXX test read 11% and 6% of samples as a suspect, while the ID.Vet test read 3% and 6% of samples as a doubtful, respectively (S2 Table). As before, in order to avoid biased seroprevalence estimation, the results for the two ELISA tests were reported under two scenarios.

**Table 2:** Differences in seroprevalence estimates from samples obtained after Schmallenberg virus (SBV) infection in two areas of Ireland (I) and in Belgium (II) using two ELISA assays and the Serum Neutralisation Test (SNT) with inclusion of suspect/doubtful in the negative results (a) and in the positive results (b).

**I.**	*Ashford*	*Rathdrum*	
**n =**	38	31	
**a) Suspect/doubtful included in the negative results**			
^*^Seroprevalence (%):			
**IDEXX**	53 (37–68)	13 (4–30)	
**ID.Vet**	76 (60–89)	87 (70–96)	
**SNT**	79 (63–90)	97 (83–100)	
**b) Suspect/doubtful included in the positive results**			
^*^Seroprevalence (%):			
**IDEXX**	63 (46–78)	19 (7–37)	
**ID.Vet**	79 (63–90)	94 (79–99)	
**SNT**	79 (63–90)	97 (83–100)	
**II.**	*ewes*	*lambs 0 hpl*	*lambs 48 hpl*
**n =**	22	36	36
**a) Suspect/doubtful included in the negative results**			
^**^Seroprevalence (%):			
**IDEXX**	77 (55–92)	3 (0–15)	64 (46–79)
**ID.Vet**	91 (71–99)	0 (0–10)	97 (85–100)
**SNT**	100 (85–100)	0 (0–10)	100(90–100)
**b) Suspect/doubtful included in the positive results**			
^**^Seroprevalence (%):			
**IDEXX**	95 (77–100)	3 (0–15)	86 (70–95)
**ID.Vet**	95 (77–100)	0 (0–10)	100 (90–100)
**SNT**	100 (85–100)	0 (0–10)	100 (90–100)

* Seroprevalence is shown for serum samples from two East Wicklow areas (Ashford and Rathdrum).

** Seroprevalence for samples from Belgian ewes and their lambs (at day 0 and 48 hours post-lambing (hpl)). For every assay, the 95% confidence exact interval (CI) is indicated between the brackets.

Firstly, all suspect/doubtful reads were considered to be negative results. A seroprevalence of 53% (CI = 37%-68%) and 13% (CI = 4–30) for the Ashford and Rathdrum areas was measured with the IDEXX test, respectively. For the ID.Vet test, a higher seroprevalence of 76% (CI = 60%-89%) and 87% (CI = 70%-96%) was observed for two areas, respectively (Table 2, part Ia).

Secondly, all suspect/doubtful reads were considered to be positive results. For the IDEXX test this resulted in a seroprevalence of 63% (CI = 46%-78%) and 19% (7%-37%) in Ashford and Rathdrum, respectively. The ID.Vet test recorded a seroprevalence of 79% (CI = 63%-90%) and 94% (CI = 79%-99%), respectively (Table 2, part Ib).

Noticeable differences between the sensitivity of the three assays and the seroprevalence of the sheep flocks were found. These dissimilarities were sharper with samples collected in the Rathdrum area. By using the IDEXX test, SBV infection in Ireland was demonstrated, but strikingly, in the epidemiological context, a noticeable lower prevalence was recorded in the Rathdrum area compared to the Ashford area (Table 2, part I). Data obtained from both ELISA tests and SNT were plotted together to better assess differences and concordances between the assays (Fig 1B and 1C).

The second field context consisted of sheep serum samples from Belgium. To analyse the performance of the SNT, IDEXX, and ID.Vet tests, serum from 22 ewes and 36 of their lambs were selected.

For ewes, the SNT read all the samples as positive (CI = 85%-100%), while IDEXX and ID.Vet tests read 18% and 5% samples as suspect/doubtful, respectively. As in the previous field context, in order to avoid bias seroprevalence estimation, the results for the two ELISA tests were reported under two scenarios. After all the suspect/doubtful samples were included in the negative results, the seroprevalence for the IDEXX and the ID.Vet test was 77% (CI = 55%-92%) and 91% (CI = 71%-99%), respectively (Table 2, part IIa). That changed in the second scenario to a seroprevalence of 95% (77%-100%) (Table 2, part IIb).

For lambs at 0 hpl, the SNT showed 36/36 samples as negative, which was expected due to the lack of antibody transfer through ruminant placenta [18]. The IDEXX test however recognized 35/36 with one false positive and the ID.Vet test recognized 36/36 as negatives, with none suspect/doubtful samples (S3 Table) (Table 2, part II).

Furthermore, knowing that all of the lambs received colostrum from positive ewes after 48 hours, it was expected from the tests to read all samples from 48 hpl as positives. Only the SNT recognized 100% (CI = 92%-100%) of samples as positives (Table 2, part II). The IDEXX test and the ID.Vet test read 22% and 3% of samples as suspect/doubtful, respectively. In order to avoid biased seroprevalence estimations, the results of the two ELISA test for lamb samples collected 48 hpl were reported under two scenarios. When suspect/doubtful were included in the negative results the seroprevelance for the IDEXX and the ID.Vet test was 64% (CI = 46%-79%) and 97% (CI = 85%-100%), respectively (Table 2, part IIa). However, when suspect/doubtful were included in the positive results, the seroprevelance for the IDEXX and the ID.Vet test was 86% (CI = 70%-95%) and 100% (CI = 90%-100%), respectively (Table 2, part IIb).

The IDEXX test showed a diminished possibility for recognizing SBV antibodies in both ewe and lamb 48 hpl samples. Data obtained from Belgium samples, from both ELISA tests and SNT were plotted together to better assess these differences (Fig 1D). Plotted results showed sensitivity differences for ewe and lamb samples between the IDEXX test and the SNT. The overall observation, once again, showed that the ID.Vet test is more sensitive than IDEXX, with results closer to the SNT, especially when lamb sera were tested (Table 2, part II).

### Performance and concordance of ELISA tests

To better assess the estimates of diagnostic performances and agreements between ELISAs and SNT as the non-reference standard, the positive percentage of agreement (PPA), negative percentage of agreement (NPA), and Cohens’s Kappa were calculated. In order to avoid biased estimation of diagnostic performance, the analyses were conducted in two scenarios by including suspect/doubtful ELISA readings, either as negative or as positive results (Table 3).

**Table 3:** Diagnostic differences between ELISA tests used for samples collected in Ireland (April 2013) and in Belgium (September 2016) with inclusion of suspect/doubtful in the negative results (a) and in the positive results (b).

**a) Suspect/doubtful included in the negative results**					
			**PPA (%)**	**NPA (%)**	^**a**^ **κ**
**Ireland**		**IDEXX**	37 (25–50)	78 (40–96)	0.05 (0.06)
		**ID.Vet**	88 (77–95)	67 (31–91)	0.46 (0.14)
**Belgium**	**Ewes**	**IDEXX**	77 (54–91)	NA	NA
		**ID.Vet**	91 (69–98)	NA	NA
	**Lambs**	**IDEXX**	64 (46–79)	97 (84–100)	0.61 (0.08)
		**ID.Vet**	97 (84–100)	100 (88–100)	0.97 (0.12)
**b) Suspect/doubtful included in the positive results**					
			**PPA (%)**	**NPA (%)**	^**a**^ **κ**
**Ireland**		**IDEXX**	47 (34–60)	78 (40–96)	0.10 (0.07)
		**ID.Vet**	95 (83–98)	67 (31–91)	0.57 (0.14)
**Belgium**	**Ewes**	**IDEXX**	95 (75–100)	NA	NA
		**ID.Vet**	95 (75–100)	NA	NA
	**Lambs**	**IDEXX**	86 (70–95)	97 (94–100)	0.83 (0.06)
		**ID.Vet**	100 (88–100)	100 (88–100)	1.00 (0.02)

Abbreviations: PPA—positive percentage of agreement, NPA—negative percentage of agreement, κ - Cohen’s Kappa

^a^ For each κ value, the standard error is indicated between the brackets. NA—an error if the calculations could not be achieved due to the properties of the tested population.

For the samples collected in Ireland, in the first scenario, the IDEXX test results showed PPA and NPA of 37% (CI = 25%-50%) and 78% (CI = 40%-96%), respectively, and no test agreement of 0.05. The ID.Vet test results showed PPA of 88% (CI = 77%-95%) and NPA of 67% (CI = 31%-91%) with weak level of agreement of 0.46 (Table 3A). The second scenario showed changes in PPA and κ values for both ELISA tests. PPA of 47% (CI = 34%-60%) and 95% (CI = 83%-98%) and higher levels of agreement of 0.10 and 0.57 were obtained for the IDEXX and ID.Vet test, respectively (Table 3B).

For the Belgian samples, two populations (ewes and their lambs) were tested according to two possible scenarios. In the first scenario for ewes, PPA values of 77% (CI = 554%-91%) and 91% (CI = 69%-98%) were recorded for the IDEXX and ID.Vet test, respectively (Table 3A). The second scenario showed increased PPA values for both tests of 95% (CI = 75%-100%), respectively (Table 3B). However, due to the properties of the tested population, in both scenarios it was not possible to calculate NPA and κ agreement.

For samples collected from lambs, the PPA and NPA values for the IDEXX test in the first scenario were 64% (CI = 46%-79%) and 97% (CI = 84%-100%), respectively. In addition, a moderate agreement with SNT of 0.61 was recorded (Table 3A). In the second scenario the PPA values increased to 86% (CI = 70%-95%), with the strong level of agreement, measuring 0.83 (Table 3B).

However, in both scenarios the ID.Vet test scored better, showing PPA of 97% (CI = 84%-100%) and NPA of 100% (CI = 88%-100%), and almost perfect agreement of 0.97 for the first scenario. In the second scenario, PPA and NPA of 100% (CI = 88%-100%) and perfect agreement of 1 for were measured for the ID.Vet test (Table 3). These results demonstrated that overall, the ID.Vet test is more likely to correctly detect samples with SBV antibodies, with almost perfect level of agreement with SNT.

### Theoretical sample size calculation for SBV detection in different seroprevalence

**Fig 2:**
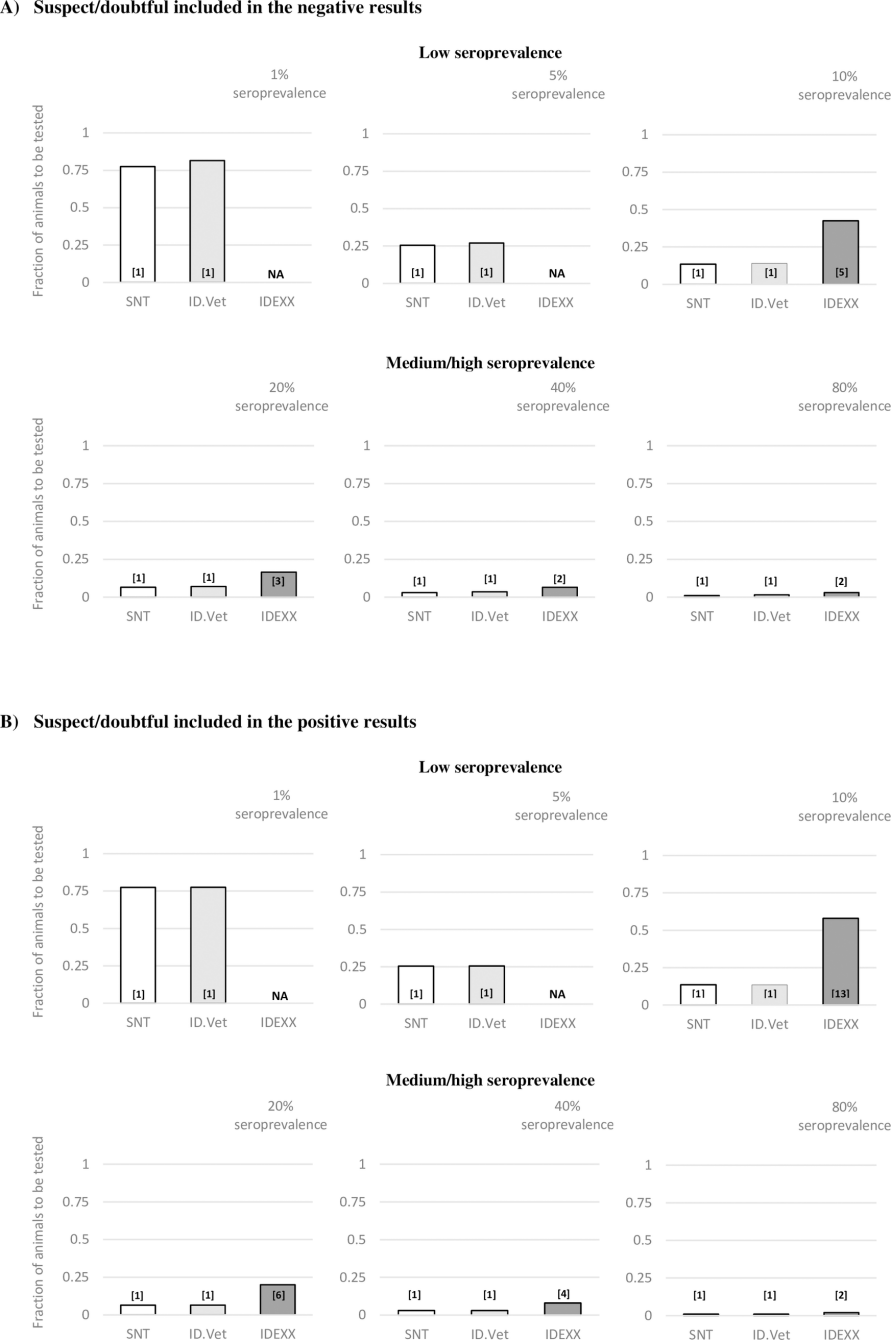
The theoretical fraction of random animals required to be tested from finite sheep flock (n = 200) in order to demonstrate that the population is SBV infected. Calculations with sensitivities and specificities (Se/Sp) obtained from total control testing from two scenarios: **(A)** all suspect/doubtful reads were included in positive results, **(B)** all suspect/doubtful reads were included in negative results. The results for the given theoretical prevalence are shown for Serum Neutralisation Test (SNT), ID.Vet test and IDEXX test. A minimum number of positive animals needed to be recognised from all tested animals in order to conclude that the population is SBV infected is indicated in square brackets for each test. NA—an error if the calculations could not be achieved within the limits of the population and/or maximum sample size.

Since different assay abilities were identified in the controlled conditions (Table 1), their impact in different theoretical seroprevalence situations was addressed. The number of random animals needed to be screened with the tests with imperfect specificities and sensitivities in different seroprevalence situations was assessed within a finite size sheep flock (N = 200) (Fig 2). In order to avoid biased estimation of the sensitivity and specificity, the results were reported under two scenarios. In the first scenario, all suspect/doubtful reads were included in negative results (Fig 2A), while in the second scenario, all suspect/doubtful reads were included in positive results (Fig 2B).

In the hypothetical situation, SNT, with reported specificity (Sp) and sensitivity (Se) of 100% for both scenarios, was able to establish flock disease in all tested seroprevalence situations (1%-80%). For SNT, in low seroprevalence of 1%, 5% and 10%, 155, 51 and 27 animals from the finite flock should be tested and only one or more should be screened positive to conclude certainly that the theoretical flock is SBV infected (Fig 2A and 2B). Results for the ID.Vet test closely followed the SNT calculations, except for a difference in the total number of animals needed to be tested in the first scenario. Here, in 1%, 5% and 10% of seroprevalence, 163, 54 and 28 animals should be tested with ID.Vet test, respectively, and one or more should be screened positive to conclude that the theoretical flock is SBV infected (Fig 2A). However, for the IDEXX test it was not possible to calculate theoretical values for seroprevalence of 1% and 5%, in both scenarios, due to the limits of the population tested (Fig 2A and 2B). For seroprevalence of 10%, in the first scenario, IDEXX test needed 85 random animals to be tested and 5 or more should be positive to determine if the flock is SBV infected (Fig 2A). In the second scenario, the number was higher, with 116 random animals needed to be tested and 13 or more should be positive to determine infection in the flock (Fig 2B).

In higher theoretical seroprevalence situations of 20%, 40% and 80%, SNT needed 13, 6 and 2 animals tested. For the first scenario, the ID.Vet test needed 14, 7 and 3 animals tested, with one or more screened positive to conclude that SBV infection is in the flock (Fig 2A). In the second scenario, the ID.Vet test had the same results as the SNT (Fig 2B). On the other side, for the IDEXX test in the same seroprevalence situations and the first scenario, 33, 13 and 6 animals should be tested and three, two, two or more should be positive in order to conclude that the flock is infected, respectively (Fig 2A). For the second scenario, 40, 16 and 4 random animals should be screened, with minimum 6, 4 and 2 positives discovered, respectively (Fig 2B).

## Discussion

The ability to detect new and emerging diseases is of high importance. Since its discovery in 2011, SBV spread throughout Europe and a number of studies in the recent years showed a reoccurrence of SBV infections and could give a conclusion that the virus became endemic [12, 13]. Because of the obligation in numerous EU countries to report suspected SBV cases to the competent authorities, confirmation with a reliable laboratory test is needed. It is generally accepted that the detection of viral RNA using rt-RT PCR presents the most reliable option in confirming acute SBV infection. However, due to the destruction of viral RNA (depending on the time of the sampling or the transport and storage conditions), short time of viremia or missing clinical signs in male animals, the clinical case number could be underestimated. Therefore, serological studies are needed to determine the real number of animals in the population infected by SBV [19].

In the study presented here, specificity, sensitivity and diagnostic performance of three different serological assays used in SBV antibodies detection were evaluated. Testing the sheep samples of known status with SNT, IDEXX test, and ID.Vet test showed the SNT as the best in detecting SBV antibodies and set it as a non-reference standard for further testing on the field samples. These results were uniformed with previously described situations in interlaboratory comparisons and laboratory proficiency trials. There, it was demonstrated that the SNT could serve as the standard test and had outstanding diagnostic performance, sensitivity and specificity [20, 21]. These results were in agreement with previous studies reporting high reliability of tests targeting SBV neutralising antibodies [22, 23].

Further, the results obtained from the field samples and compared in this study showed noticeable differences in the tests used to detect SBV antibodies during the 2013 lambing season in two East Wicklow areas (Ashford and Rathdrum) in Ireland and after the 2016 re-emergence of the virus in Belgium. Even in the hypothetical seroprevalence situations, assays showed different abilities to detect SBV infection in sheep population. The ELISA tests were able to detect diseased population in higher seroprevalence, from 20–99%, using a higher number of animals tested compared to SNT. However, for low theoretical seroprevalence, from 1–10%, the ELISA tests performance was inadequate to calculate a number of sheep needed to test positive to determine infected flock, which would be of importance in SBV re-emerging situations.

Discrepancies and performance drawbacks observed in the present study could be due to differences in tested antigenic aspects of the three assays involved. The SNT detects SBV antibodies raised against glycoprotein N (Gn) and glycoprotein C (Gc), and both ELISA tests are based on the ability to detect antibodies raised against the N-protein. Differences between the IDEXX and ID.Vet ELISA test sensitivity raise questions about the antigens and the antigen processing used in the assays.

First, epitopes present in the recombinant peptide used in the ID.Vet test might be absent and/or modified and/or inaccessible in the IDEXX ELISA, which could lead to lower sensitivity to SBV antibodies. Second, differences in the individual tests might be connected to (i) the differences in immunogenicity of the peptides used in the kits, (ii) the accessibility of the peptide, (iii) tested animal species, (iv) the coating process, (v) the reagents used and manufacturing process. In the Ashford area, both ELISA tests were able to detect antibodies raised against the N protein. However, in Rathdrum, the IDEXX test failed to do so. Coupeau *et al*., 2016 [24] showed the diversity of the SBV viral population within a single sheep flock. Sheep infected with SBV in 2011 obtained a high level of seroprevalence, however, SBV re-emergence occurred in 2012 [14] and again in 2016. Finally, the ELISA manufacturers based their calculations on SBV antibody detection in ruminant serum or plasma from multiple species, including cattle and goats. Altogether, these test-specific differences could be the reason for the discrepancies in the results and test performances.

Finally, models of the assay performance in different seroprevalence situations showed different adaptations of the three tests for monitoring SBV infection. In massive infection and high seroprevalence, the three assays in question were able to detect SBV infection by testing different numbers of random animals in the population. In programs for monitoring freedom of disease in sheep population, the IDEXX ELISA test was not able to recognise SBV infection, leaving only SNT and ID.Vet test adapted to provide results.

Many studies showed that SNT could be used as a reference test to determine the true status of sheep flock infected by SBV and to confirm results obtained with commercially available ELISA tests. For surveillance and monitoring programs, SNT is best adapted to detect SBV antibodies in low seroprevalence situations. However, time-consuming manipulations and the long period of incubation (four days or more) to obtain results make it impracticable, especially for monitoring and surveillance studies. Our results showed that ID.Vet ELISA test, with almost perfect agreement with SNT, could be used as an appropriate substitution for Serum Neutralisation Test.

## Conclusion

Surveillance programs for emerging and re-emerging diseases need reliable and sensitive tests, especially in low seroprevalence situation. In this study, Schmallenberg Virus (SBV) was used as a model to evaluate the performance, and to select the best-adapted test for antibody detection according to the seroprevalence level of an emerging virus in sheep. In massive infection and high seroprevalence situations, the three assays in question are able to detect SBV infection by testing different numbers of random animals in the population. However, in programs for monitoring freedom of disease in sheep population, the IDEXX test is not adapted to detect SBV infection at the population level when seroprevalence is low, leaving SNT and ID.Vet ELISA as the only adapted tests.

## Supporting information

S1 Table Cross-tabulation of data obtained from control samples for different tests. (DOCX)

S2 Table Cross-tabulation of data obtained from samples collected from two areas in Ireland for different tests. (DOCX)

S3 Table Cross-tabulation of data obtained from samples collected from ewes and lambs (0 hpl and 48 hpl) in Belgium for different tests. (DOCX)
