# Supplementary material for: Test selection for antibody detection according to the seroprevalence level of Schmallenberg virus in sheep
Source: PLoS One. 2018 Apr 27;13(4):e0196532. doi: 10.1371/journal.pone.0196532 (PMC5922541; doi:10.1371/journal.pone.0196532)
Supplement: S1 Table — (DOCX) [file pone.0196532.s001.docx]

**S1 Table.** Cross-tabulation of data obtained from control samples for different tests.

| 1. **Serum Neutralisation Test (SNT)** | ***Test*** | ***Disease*** | | | | ***Suspect/ doubtful*** |
| --- | --- | --- | --- | --- | --- | --- |
|  |  | *Present* | | *Absent* | |  |
|  | *Positive* | True + | 45 | False + | 0 | 0 |
|  | *Negative* | False - | 0 | True - | 45 | 0 |
| 1. **IDEXX ELISA** | ***Test*** | ***Disease*** | | | | ***Suspect/ doubtful*** |
|  |  | *Present* | | *Absent* | |  |
|  | *Positive* | True + | 35 | False + | 1 | 7 |
|  | *Negative* | False - | 3 | True - | 42 | 2 |
| 1. **ID.Vet ELISA** | ***Test*** | ***Disease*** | | | | ***Suspect/ doubtful*** |
|  |  | *Present* | | *Absent* | |  |
|  | *Positive* | True + | 43 | False + | 0 | 2 |
|  | *Negative* | False - | 0 | True - | 45 | 0 |
