# Supplementary material for: Test selection for antibody detection according to the seroprevalence level of Schmallenberg virus in sheep
Source: PLoS One. 2018 Apr 27;13(4):e0196532. doi: 10.1371/journal.pone.0196532 (PMC5922541; doi:10.1371/journal.pone.0196532)
Supplement: S2 Table — (DOCX) [file pone.0196532.s002.docx]

**S2 Table.** Cross-tabulation of data obtained from samples collected from two areas in Ireland for different tests.

| 1. **Ashford** | | | | | | |
| --- | --- | --- | --- | --- | --- | --- |
| 1. **Serum Neutralisation Test (SNT)** | ***Test*** | ***Disease*** | | | | ***Suspect/ doubtful*** |
|  |  | *Present* | | *Absent* | |  |
|  | *Positive* | True + | 30 | False + | 0 | 0 |
|  | *Negative* | False - | 0 | True - | 8 | 0 |
| 1. **IDEXX ELISA (compared do SNT)** | ***Test*** | ***Disease*** | | | | ***Suspect/ doubtful*** |
|  |  | *Present* | | *Absent* | |  |
|  | *Positive* | True + | 18 | False + | 2 | 4 |
|  | *Negative* | False - | 8 | True - | 6 | 0 |
| 1. **ID.Vet ELISA (compared do SNT)** | ***Test*** | ***Disease*** | | | | ***Suspect/ doubtful*** |
|  |  | *Present* | | *Absent* | |  |
|  | *Positive* | True + | 27 | False + | 2 | 1 |
|  | *Negative* | False - | 2 | True - | 6 | 0 |
| 1. **Rathdrum** | | | | | | |
| 1. **Serum Neutralisation Test (SNT)** | ***Test*** | ***Disease*** | | | | ***Suspect/ doubtful*** |
|  |  | *Present* | | *Absent* | |  |
|  | *Positive* | True + | 30 | False + | 0 | 0 |
|  | *Negative* | False - | 0 | True - | 1 | 0 |
| 1. **IDEXX ELISA (compared do SNT)** | ***Test*** | ***Disease*** | | | | ***Suspect/ doubtful*** |
|  |  | *Present* | | *Absent* | |  |
|  | *Positive* | True + | 4 | False + | 0 | 2 |
|  | *Negative* | False - | 24 | True - | 1 | 0 |
| 1. **ID.Vet ELISA (compared do SNT)** | ***Test*** | ***Disease*** | | | | ***Suspect/ doubtful*** |
|  |  | *Present* | | *Absent* | |  |
|  | *Positive* | True + | 26 | False + | 1 | 2 |
|  | *Negative* | False - | 2 | True - | 0 | 0 |
