# Supplementary material for: Test selection for antibody detection according to the seroprevalence level of Schmallenberg virus in sheep
Source: PLoS One. 2018 Apr 27;13(4):e0196532. doi: 10.1371/journal.pone.0196532 (PMC5922541; doi:10.1371/journal.pone.0196532)
Supplement: S3 Table — (DOCX) [file pone.0196532.s003.docx]

**S3 Table.** Cross-tabulation of data obtained from samples collected from ewes and lambs (0 hpl and 48 hpl) in Belgium for different tests.

| 1. **Ewes** | | | | | | |
| --- | --- | --- | --- | --- | --- | --- |
| 1. **Serum Neutralisation Test (SNT)** | ***Test*** | ***Disease*** | | | | ***Suspect/ doubtful*** |
|  |  | *Present* | | *Absent* | |  |
|  | *Positive* | True + | 22 | False + | 0 | 0 |
|  | *Negative* | False - | 0 | True - | 0 | 0 |
| 1. **IDEXX ELISA (compared do SNT)** | ***Test*** | ***Disease*** | | | | ***Suspect/ doubtful*** |
|  |  | *Present* | | *Absent* | |  |
|  | *Positive* | True + | 17 | False + | 0 | 4 |
|  | *Negative* | False - | 1 | True - | 0 | 0 |
| 1. **ID.Vet ELISA (compared do SNT)** | ***Test*** | ***Disease*** | | | | ***Suspect/ doubtful*** |
|  |  | *Present* | | *Absent* | |  |
|  | *Positive* | True + | 20 | False + | 0 | 1 |
|  | *Negative* | False - | 1 | True - | 0 | 0 |
| 1. **Lambs (0 hpl and 48 hpl)** | | | | | | |
| 1. **Serum Neutralisation Test (SNT)** | ***Test*** | ***Disease*** | | | | ***Suspect/ doubtful*** |
|  |  | *Present* | | *Absent* | |  |
|  | *Positive* | True + | 36 | False + | 0 | 0 |
|  | *Negative* | False - | 0 | True - | 36 | 0 |
| 1. **IDEXX ELISA (compared do SNT)** | ***Test*** | ***Disease*** | | | | ***Suspect/ doubtful*** |
|  |  | *Present* | | *Absent* | |  |
|  | *Positive* | True + | 23 | False + | 1 | 8 |
|  | *Negative* | False - | 5 | True - | 35 | 0 |
| 1. **ID.Vet ELISA (compared to SNT)** | ***Test*** | ***Disease*** | | | | ***Suspect/ doubtful*** |
|  |  | *Present* | | *Absent* | |  |
|  | *Positive* | True + | 35 | False + | 0 | 1 |
|  | *Negative* | False - | 0 | True - | 36 | 0 |
